# Ghrelin and Metabolic Surgery

**DOI:** 10.1155/2010/217267

**Published:** 2010-01-27

**Authors:** Dimitrios J. Pournaras, Carel W. le Roux

**Affiliations:** ^1^Department of Bariatric Surgery, Musgrove Park Hospital, Taunton, Somerset TA1 5DA, UK; ^2^Imperial Weight Centre, Imperial College London, London W6 8RF, UK

## Abstract

Metabolic surgery is the most effective treatment for morbid obesity. Ghrelin has been implicated to play a role in the success of these procedures. Furthermore, these operations have been used to study the gut-brain axis. This article explores this interaction, reviewing the available data on changes in ghrelin levels after different surgical procedures.

## 1. Introduction

Surgical procedures are currently the most effective therapy for long-term weight loss [1]. Furthermore, some of these operations lead to the rapid remission of type 2 diabetes in a weight loss independent manner [2]. The mechanism that leads to sustained weight loss as well as diabetes remission after bariatric operations remains to be fully elucidated. However, it is becoming evident that these procedures modulate the gut-brain axis by altering the anatomy of the gut and affecting gut hormones [3]. In fact some of these procedures are now considered suitable models to study the gut brain axis. The landmark study of Cummings et al., which showed a profound suppression of ghrelin levels following Roux-en-Y gastric bypass, has brought the interaction of weight loss operations and the gut-brain axis into focus [4–6].

### 1.1. Ghrelin

Ghrelin is a 28-amino acid peptide produced from the fundus of the stomach and the proximal intestine [5–7]. It is the only known orexigenic gut hormone. Central and peripheral administration leads to increased food intake [8, 9]. Ghrelin levels increase prior to meals and are suppressed postprandially in proportion to the amount of calories ingested, therefore suggesting a possible role in meal initiation [10, 11]. The 24-hour profile of ghrelin increases following diet-induced weight loss supporting the hypothesis that ghrelin has a role in the long-term regulation of body weight [4]. Obese individuals have lower fasting ghrelin levels, and significantly reduced postprandial ghrelin suppression compared to normal weight individuals [12].

### 1.2. Metabolic Surgery

Surgical procedures were designed to promote weight loss by reducing stomach volume (laparoscopic adjustable gastric banding (LABG), laparoscopic sleeve gastrectomy (LSG)), malabsorption of nutrients (biliopancreatic diversion (BPD), duodenal switch (DS)), or a combination of both (Roux-en-Y gastric bypass (RYGB)). It is now known that standard proximal Roux-en-Y gastric bypass causes only malabsorption of micronutrients and not of calories, and restriction does not play a major role. A number of studies have shown that changes in gut hormone concentrations may partly explain the weight loss following weight losss surgery.

Furthermore, following these procedures a vast improvement in glycaemic control has been observed. A meta-analysis reported improved glycaemic control and remission of type 2 diabetes in 83.8% of patients following gastric bypass and 47.8% following gastric banding [2]. The fact that these procedures improve manifestations of the metabolic syndrome, often in a weight-loss independent manner has led to the development of the concept of metabolic surgery. In fact a number of scientific societies have changed their name in order to include this term.

## 2. Ghrelin and Roux-en-Y Gastric Bypass

RYGB is the most common metabolic procedure worldwide [13]. Cummings et al. showed a profound suppression of ghrelin levels (24-hour profile) following RYGB [4]. However, the data published since are inconclusive. Different studies showed a decrease in fasting and postprandial ghrelin levels [14–21], no change in fasting and postprandial levels [22–32], and an increase in fasting ghrelin levels after RYGB [33–37]. The reason for this heterogeneity has not been elucidated and multiple explanations have been proposed. Differences in the methods used for evaluating ghrelin levels are possible, but unlikely. It has been suggested that even in the studies reporting an increase in fasting ghrelin levels, ghrelin does not increase to the extent reported with diet-induced weight loss or of nonobese individuals [26]. In a study which investigated the intraoperative changes in ghrelin during RYGB, the complete division of the stomach and the formation of a vertical pouch contributed to the decline in the circulating ghrelin [18]. Moreover, an intact vagus nerve appears to be required for ghrelin to have an appetite effect [38]. Technical differences in the procedure in regards to preservation of the vagus nerve may be responsible. Iatrogenic vagal nerve dysfunction caused intraoperatively might also play a role, as shown by a study which demonstrated a decrease in ghrelin levels on the first postoperative day after RYGB, followed by an increase to preoperative levels at 1 month and a further increase at 12 months [35]. An alternative theory has suggested that the different configuration of the pouch might be responsible [39]. In a vertical pouch, ghrelin producing cells are more likely to be excluded, compared to a horizontal pouch [40]. Finally, hyperisulinaemia and insulin resistances are associated with ghrelin suppression in obese individuals [40]. Therefore, preoperative differences as well as differences in the postoperative improvement in these parameters may cause this inconsistency.

## 3. Ghrelin and Biliopancreatic Diversion

BPD is an operation that does cause malabsorption of calories. A study on patients prior to and 5 days and 2 months after BPD showed a similar response with an initial reduction in fasting ghrelin, followed by a return to the preoperative levels when food consumption resumed to almost preoperative levels [41]. This finding supports the hypothesis that although the primary source of ghrelin is the gastric mucosa, small intestinal nutrient exposure is sufficient for food-induced plasma ghrelin suppression in humans and gastric nutrient exposure is not necessary for this suppression [42]. However, different studies have shown an increase [43–46] or no change [36, 41, 47] in ghrelin levels after BPD.

A decrease in ghrelin levels has been noted after DS [48, 49]. In this procedure, the reduction in the stomach volume is achieved with a sleeve gastrectomy. It is important to stress the anatomical difference between BPD as described by Scopinaro with horizontal gastrectomy where the fundus remains intact and gets in contact with nutrients, whereas in DS type duodenal switch with sleeve gastrectomy the fundus, the main area of ghrelin production, is resected.

## 4. Ghrelin and Gastric Banding

LAGB has been shown to reduce hunger and increase satiety [50]. No association with ghrelin levels was demonstrated in the same study [51]. Schindler et al. showed an increase in fasting ghrelin accompanied by a paradoxical decrease in hunger after LAGB suggesting that weight loss is independent of circulating plasma ghrelin and relies on changes in eating behaviour induced by gastric restriction [51]. Further studies on patients following LAGB demonstrated both increased fasting ghrelin levels [7, 19, 25, 52–54] and a blunted postprandial suppression of ghrelin [17, 32]. However, we, as well as others, were not able to show any changes in ghrelin levels after laparoscopic gastric banding [22, 55–57].

## 5. Ghrelin and Sleeve Gastrectomy

LSG is a relatively new bariatric operation which was designed as a restrictive procedure. Recent studies challenge this classification showing accelerated gastric emptying after LSG [58]. However, another study of patients undergoing LSG showed no difference in gastric emptying compared to preoperatively and therefore the controversy remains [59]. The fact that the fundus of the stomach, the main location of ghrelin-producing cells, is excluded in this procedure led to speculation that ghrelin could play a role in the mechanism of action. Three studies confirmed a decrease in fasting ghrelin levels after LSG [54, 57, 60]. A prospective, double-blind study comparing RYGB and LSG confirmed a significant postprandial suppression of ghrelin postoperatively, while there was no change in the RYGB group [30]. In the same study, the marked suppression of ghrelin levels after LSG was associated with greater appetite reduction and excess weight loss during the first postoperative year compared to RYGB [30]. An even more recent prospective randomised comparison of LSG and RYGB confirms that both operations reduce fasting and meal stimulated ghrelin levels, significantly more so after LSG, so resection of the fundus has more impact on the ghrelin levels compared to just bypassing it [61].

## 6. Conclusion

The role of ghrelin in the success of bariatric and metabolic surgery remains to be further elucidated. Some of the findings of the initial studies have not been confirmed in more recent investigations. Different gut hormones as well as having an incretin effect have been implicated to be key players in appetite control. However, the hypothesis that ghrelin might play a role in the mode of action of metabolic surgery has been crucial in the development of the field. The weight loss as well as the remission of type 2 diabetes experienced after metabolic surgery is not exclusively attributed to pure restriction or malabsorption any more. A lot of research is focused on exploring the hormonal and metabolic changes after metabolic surgery as well as the mechanism of action.
